# Stability of Alum-Containing Paper under Alkaline Conditions

**DOI:** 10.3390/molecules25245815

**Published:** 2020-12-09

**Authors:** Michal Jablonský, Jozef Šima

**Affiliations:** 1 Department of Wood, Pulp and Paper, Faculty of Chemical and Food Technology, Slovak University of Technology in Bratislava, Radlinského 9, SK-812 37 Bratislava, Slovakia; 2 Department of Inorganic Chemistry, Faculty of Chemical and Food Technology, Slovak University of Technology in Bratislava, Radlinského 9, SK-812 37 Bratislava, Slovakia; jozef.sima@stuba.sk

**Keywords:** alum-rosin sized paper, degradation, deacidification, homogeneity of alum

## Abstract

The present contribution evaluates the methods of degradation and stabilization of alum-containing paper with a focus on the alkaline environment achieved by deacidification procedures. In terms of reviewed subjects, the contribution focuses on alum-rosin sized paper, which is still used as a carrier of knowledge and information; however, it also mentions cellulose itself and other brands of paper. The contribution summarizes the results on the homogeneity of the distribution of alum and rosin in the paper mass and on the paper surface. It provides the knowledge gained in the field of alkaline hydrolysis and oxidation with special regard to transition metal species. It shows the values of alkaline reserves achieved in the main mass-deacidification processes. On the basis of the acquired knowledge, the contribution emphasizes the procedures of paper stabilization. Criteria of “increased mechanical permanence and lifetime prolongation” adopted to evaluate and compare the efficacy of individual mass-deacidification processes were applied and corresponding data are introduced. The contribution also draws attention to the existence of open issues in the area of paper degradation and stabilization.

## 1. Introduction

In our recent paper [1] regarding the factors influencing the degradation of cellulose in alum-rosin sized paper, five main questions were raised and tentatively answered. For the sake of completeness, the questions were as follows:

(a) How does the mechanism of hydrolytic reactions of Al(III) compounds affect pH and, thus, hydrolytic degradation?
(b) How do otherwise redox-stable Al(III) compounds participate in the redox reactions occurring in paper and, therefore, contribute to redox degradation?
(c) How can sulfuric acid and organic acids be formed by the redox reactions occurring in the paper and the reactions stimulated by nitrogen and sulfur oxides present in the environment?
(d) What is the effect of present rosin on the course of redox and hydrolytic reactions?
(e) Does the oxidation capacity of actual or potential oxidizing agents depend on pH and, if so, how?

However, some issues remain open and are discussed in this paper. The issues are the following:

1. What is the homogeneity of alum and rosin distribution in the paper and to what extent does the inhomogeneity affect the paper degradation process?
2. What is the role of alkaline hydrolysis and related oxidative processes in paper degradation?
3. What is the impact of deacidification on the stability/fragility of alum-containing paper?
4. Which levels of pH and alkaline reserve can be reached in the treated alum-containing paper?

Most research activities devoted to alum-rosin sized paper deal with processes in acidic environments. This is logical because the paper itself is acidic and one of the main approaches for its degradation is acidic hydrolysis. On the other hand, research focusing on the processes taking place in the paper in an alkaline environment has been comparatively scarce. The knowledge accumulated shows that the degradation of paper under alkaline conditions cannot be ignored. This paper focuses on the processes taking place in the paper in a basic environment.

Although the present contribution focuses on alum-rosin sized paper, for the sake of completeness, it also provides some data on pure cellulose, as its reactions may help to better understand the properties of paper and outline new applications for paper and cellulose.

## 2. Homogeneity of Alum Dispersion in Paper

Paper consists of cellulose fibers, fillers, and auxiliaries [2]. The fibers have a hydrophilic character and are wettable by water. To reduce the wettability and absorbency of the paper, the sizing process has been used for many decades. Alum-rosin sizing was invented by Moritz Friedrich Illig in Germany [3]. Generally, there are two types of sizing, internal and external. Internal sizing is achieved by introducing a sizing agent into the wet end of the paper machine, universally treating the sheet. External (surface) sizing is generally applied at the size press or later to treat the sheet surface.

To answer the first question concerning the homogeneity of the distribution of alum and rosin in the paper and its consequences for the paper degradation process, a few measurements of the homogeneity achieved at internal and external sizing have been performed.

The influence of papermaking conditions on the distribution of rosin and alum was evaluated in detail and described by Wang et al. [4]. Logically, it could be assumed that, if in the papermaking process, the alum and rosin together with the cellulose form part of the cellulosic solution, the rosin and alum should be distributed homogeneously in the paper (internal sizing process in the paper).

However, Wang and his group found that rosin was unevenly distributed on the fiber surfaces and its distribution correlated to that of alum [4]. These results are consistent with an assumption that the rosin particle adsorption was controlled, at least in part, by the distribution of alum species on the fiber surfaces. In turn, the distribution of alum can be affected by factors such as refining, the existence of fines, and the possible inadequacy of mixing procedures employed during alkaline papermaking conditions. Kitaoka et al. [5] studied the way rosin, alum, and paper fibers interact. Sizing levels, as well as rosin retention ratios of paper by tub sizing with the anionic emulsion sizes, increased with increasing alum content in the base paper sheets.

In an external sizing system, rosin (mainly abietic acid or its saponification product) provides the hydrophobic property. Alum helps bond rosin onto the fibers and, as such, is called a mordant [5]. Details on the paper-making process are given elsewhere [6,7,8].

By monitoring the properties of the alum-rosin sized paper, it was found that alum is responsible for the gradual degradation of the paper since its reactions lower the pH of the paper and promote degradation via acid hydrolysis. A systematic study of the structure of the paper revealed that the properties of the paper depend significantly on the homogeneity of the alum and rosin dispersion in the paper. The issue of homogeneity also concerns deacidification substances, as heterogeneity can lead to different degrees of protective processes. The homogeneity of the alum distribution in the paper is usually observed by energy-dispersive spectroscopy (EDS). The potential of EDS was illustrated by Özgörüş et al. (2017) [9] using the example of a part of a damaged Koran folio. In the surface sizing process, a homogeneous liquid sizing mixture passes through the first fibers of the paper. Then, after multiple surface sizing, a layer is formed on the paper. SE-SEM (secondary electron imaging performed by scanning electron microscopy) clearly distinguishes between sizes of fibers, documenting the formation of sandwich layers [9]. The results showed that negligible or no Al was detected in the paper fiber, while a higher amount of Al was detected in the sizing. Damage to the alum and rosin layer depends on the case and there are not enough data available to perform a statistical evaluation of the distribution or damage.

**Figure 1:**
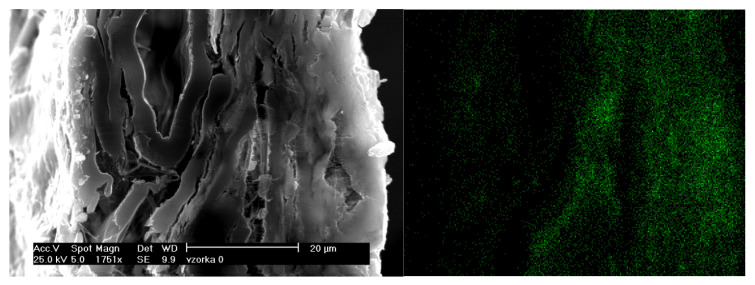
Scanning electron microscopy (SEM) images and elemental mapping of Al(III) (green dots) in cross-section of paper (acidic test paper using alum rosin sizing, grammage 45 g/m^2^, cold extract pH: 4.5–5.0, surface pH = 5.6 ± 0.3, containing bleached mechanical groundwood (55%), bleached sulfite pulp (20%), recycled fibers (15%), and clay (10%)) [10].

**Figure 2:**
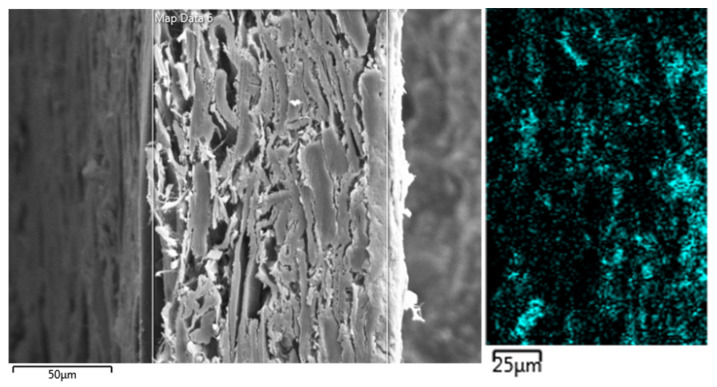
Scanning electron microscopy (SEM) images and elemental mapping of Al(III) (blue dots) in cross-section of paper (NOVO paper (Klug-Conservation, Immenstadt, Germany), resin sizing with alum; surface pH 4.5; grammage of 90 g/m^2^; composition: groundwood pulp (50–65%), bleached kraft pulp (25–35%), China clay (12–15%) [10].

Figure 1 and Figure 2 show scanning electron microscopy (SEM) images for two types of paper, namely, acidic test paper containing alum rosin sizing and standard test paper (NOVO paper) also containing alum and a distribution of Al(III) in its cross-section [10].

As can be seen, a homogeneous distribution of Al(III) in the cross-section of the paper is observable in both paper samples. NOVO paper is a standard paper used in the process of checking the effectiveness of deacidification processes (supplier KLUG Conservation). In the case of the second type of paper, it was used as a standard paper for testing and comparative evaluation of the effectiveness of commercial deacidification systems within the project “Preservation, Stabilization, and Conservation of Traditional Carriers of Information in the Slovak Republic” [11].

Figure 1 and Figure 2 show that the paper contained Al(III) homogeneously dispersed throughout the paper mass, which is a consequence of the internal sizing of the paper [10].

It is evident that, in the case of internal sizing, a homogeneous distribution of Al(III) in the paper is achieved, and, in the case of surface sizing, separate layers containing rosin and Al(III), fibers, and again rosin and Al(III) are formed in the paper.

Al(III), whether homogeneously or inhomogeneously distributed in the paper (in cross-section), can cause a deeper degradation of the cellulose due to its catalytic effect. The effect on the kinetics of cellulose degradation was described in more detail in Jablonsky et al. [1].

In conclusion, the question of whether the inhomogeneity in the distribution of rosin and alum causes differences in paper local degradation has not been sufficiently (if at all) studied and, thus, still has not been answered.

## 3. Cellulose and Paper Degradation by Alkaline Hydrolysis

Alkaline hydrolysis of cellulose plays a very important role only if cellulose contains oxidized groups. In this case, β-alkoxy elimination takes place, leading to cellulose chain depolymerization [12]. When dealing with alkaline hydrolysis, a distinction must be made between the hydrolytic reactions of pure cellulose and those of paper, particularly alum-rosin sized paper. Alkaline degradation of cellulose was investigated in both strongly alkaline systems with a pH 13 ± 1 [13,14,15] and mild alkaline systems with a pH 8–9 ± 1 [11,16,17,18]. The first case concerned the degradation of cellulose as a component of radioactive waste from nuclear installations [13,14,15]. Cellulose degradation was monitored for a long time (up to some years) at a temperature above 50 °C in cement pore water with pH ≥ 12.5. In addition to low-molecular-weight organic acids, two diastereoisomers of isosaccharinic acid were identified as major products of anaerobic degradation. On the basis of detailed kinetic and analytical measurements, the cellulose degradation under the mentioned conditions could be described as consisting of three parts: (1) the endwise clipping of glucose units through the peeling-off process, induced by a nucleophile addition of hydroxide ions to reducing end groups; (2) mid-chain scissions of the cellulose chain induced by stochastic deprotonation of glucose subunits at any place of the cellulose chain; (3) formation of non-reacting end groups through the chemical transformation of reducing end groups to *meta*-saccharinate, which stops the peeling-off process [13,14].

The chief process in alkaline hydrolysis at mild alkaline conditions is cellulose degradation from the reducing end of its molecule and hydrolysis of glycosidic bonds. Both processes occur without a considerable change in the degree of polymerization. In general, the alkaline degradation of cellulose involves also isomerization, β-alkoxy elimination (“peeling reaction”), tautomerization, and benzilic acid rearrangement to glucoisosaccharinic acid [16,17,18]. Commonly identified low-molecular-weight degradation products are formic acid, acetic acid, derivatives of propanoic, butanoic, glyceric, and lactic acids, etc.

The literature on paper degradation caused by hydrolysis and on paper stabilization is overwhelmingly devoted to acid hydrolysis occurring mainly in alum-rosin sized paper and its suppression by deacidification processes. At present, this type of paper is already produced and used to a lesser extent in hydrolysis [11,18].

It should, however, be mentioned that, in addition to acid hydrolysis, which is considered to be a key cause and mode of paper degradation, there is also a less described alkaline degradation, which is frequently associated with oxidative degradation processes. An alkaline environment is formed as a consequence of deacidification. Alkaline hydrolytic processes can contribute to the paper degradation because hydroxides, oxides, or alcoholates are used in various deacidification techniques [16,17,18]. However, it should be noted that, upon exposure to air, they rapidly transform to the corresponding carbonates, which do not induce cellulose depolymerization, i.e., paper degradation processes. When the time factor is taken into account, the period after deacidification, when the paper is slightly alkaline, is too short to consider the significant effect of alkaline hydrolysis on the stability of the paper [17,18].

Nevertheless, paper degradation by alkaline hydrolysis should not be rejected without experimental evidence of its effect. Ahn et al. [18] compared the accelerated aging of untreated acidic (alum-rosin sized) book paper with that of deacidified paper having an alkali reserve. They found that degradation rates of the original book papers were significantly reduced after mass deacidification compared to the non-deacidified duplicates. The beneficial effect of retarded hydrolytic degradation by mass deacidification, thus, clearly outweighed the possible negative alkalinity effects of the deposited alkaline reserve.

The risks of alkaline degradation during deacidification of paper oxidized by ultraviolet (UV)-A radiation and H_2_O_2_ or NaIO_4_ were evaluated by Stephens et al. [19]. Up to pH 10, the paper oxidized by UV-A radiation and H_2_O_2_ showed minimum sensitivity to the β-elimination reaction. Such a paper simulates naturally oxidized papers. Contrarily, the paper oxidized by UV-A radiation and NaIO_4_ was markedly degraded at pH 10–12.5. It seems worth mentioning that β-elimination itself is a redox process; however, its dependence on pH suggests that hydrolytic reactions are involved in the mechanism. As in the case of acid hydrolysis, alkaline degradation, thus, coincides with oxidative degradation [15,20,21].

## 4. Oxidation of Cellulose and Paper in an Alkaline Environment

In our previous contribution [22], oxidation of cellulose and paper in acidic conditions (especially of alum-rosin sized paper) or neutral media was reviewed. However, an alkaline environment also exists in “acidic paper” as a result of its deacidification and the formation of an alkaline reserve in it. This section is devoted to the oxidation of cellulose and paper in an alkaline environment.

In general, the oxidative degradation of paper results from the action of oxidizing agents (especially O_2_ and reactive oxygen species (ROS)), radiation, and the catalytic action of transition metal species (TMS). Common ROS are free singlet oxygen molecule ^1^O_2_, superoxide anion radical O_2_^•−^, its conjugated acid hydroperoxyl radical HOO^•^, hydrogen peroxide H_2_O_2_, and hydroxyl radical HO^•^. While considerable attention is paid to the first two factors, the elucidation of the function of TMS has essentially been limited to the Fenton reaction and iron gall inks.

A comparison of the oxidative and photooxidative degradation of paper deacidified by several methods applied in a mass scale was published Dufour and Havermans in 2001 [23]. In the presence of oxygen and UV radiation, deacidified (i.e., slightly alkaline) paper undergoes degradation of β-glycosidic bonds by alkaline hydrolysis and (photo)oxidation converting OH-groups to CO- and COOH-groups, thus contributing to paper yellowing. In papers containing lignin (newsprint), yellowing is also due to lignin oxidation. Regarding the deacidification and yellowing of the paper, at least two questions waiting to be answered should be mentioned. The first concerns yellowing, where the literature is still contradictory about the molecular reasons for yellowing and brightness reversion of pure (lignin- and hemicellulose-free) celluloses [24,25]. The second relates to the distribution of the deacidifying agent in the paper volume. Especially when using a solid deacidifying agent, it concentrates on the paper surface. Thus, the surface is basic, but the inner part remains acidic. As a result, outer (surface) and inner parts of the paper may be subject to different degradation reactions or the same reactions to different degrees.

Analogously to oxidation in acidic and neutral media, oxidation in alkaline media is accompanied by hydrolysis (in this case, alkaline). While the mechanism of hydrolysis is relatively well known and established in the literature [26,27,28], the oxidation of cellulose by a radical mechanism initiated by ROS is a complex process with a poorly understood mechanism that has yet to be investigated [24].

Probably the first work experimentally monitoring and theoretically elucidating the oxidation of cellulose in an alkaline medium was the paper published in 1950 [29] dealing with the oxidation by hydrogen peroxide in a strongly alkaline solution. The authors proposed mechanisms of the oxidation and identified, after a rather complicated procedure, d-arabonic acid as its main product.

This contribution is devoted to oxidation in an alkaline environment. As for the studied objects, the oxidation of pure cellulose (or its low-molecular-weight analogues imitating the properties of cellulose) and paper was studied. Regarding cellulose oxidation processes, those caused and/or catalyzed by “artificial” oxidizing agents such as the 2,2,6,6-tetramethylpiperidine-1-oxyl (TEMPO) radical, periodates, and hydrogen peroxide were followed [30,31,32,33], along with the processes involving atmospheric oxygen and corresponding ROS [34].

Most studies were devoted to thermal oxidation, but there were also works dealing with photochemical oxidation. The stimulus for investigation of photochemical degradation is the fact that most paper-based documents are usually exposed to visible and UV-A spectra during their normal use. Even despite the restrictions in archives, libraries, and museums aimed at protecting papers against light sources, the effects of the photo deterioration of paper should not be fully neglected [23,35,36,37,38]. Recent research showed, for example, that papers after deacidification became more sensitive toward photodegradation [38]. Generally, the photodegradation mechanisms of cellulose and paper can occur in the form of either direct photolysis or photosensitized processes [38,39]. Direct photolysis of cellulose and paper may be produced by wavelengths lower than 340 nm. Within direct photolysis, usually involving radicals, the degree of polymerization decreases, and low-molecular-wright products are formed. This method is practically out of the question in libraries and archives. Photosensitized degradation may be promoted by the presence of chromophores of cellulose chains, impurities, fillers, or lignin. The likelihood of such degradation in libraries and archives is very low because the energy required to break a bond in the cellulosic molecules is significantly higher than the energy of primary photoexcitation by the radiation present in the mentioned institutions. Of course, in specific cases, UV-B and UV-C radiation can contribute to cellulose degradation. This situation can be exemplified by the production of nanofibrils at the oxidation of cellulose by the attack of ozone, UV radiation, and hydrogen peroxide performed in the pH interval 2.7–9. The role of UV radiation is to generate HO^•^ radicals and H_2_O_2_ molecules [40]. However, such cases do not concern paper documents in libraries and archives.

When discussing the effect of TMS on degradation, two situations mirroring the differences in TMS concentration need to be distinguished. In the paper mass, the concentration of TMSs is rather low; TMSs are evenly distributed throughout the paper volume and, thus, their impact is also expected to be rather low. In holographs, drawings, and other historical documents written, signed, or stamped by iron gall ink, the local concentration of TMS (iron) is high and their degradation effect significant [41,42,43]. This is why many archives and collections have to face stability problems with such documents, in which the parts not containing ink are relatively stable, but the parts in which the ink has penetrated the paper mass are fragile, prone to disintegration. The impact of iron gall inks on paper is reviewed elsewhere [41,42,43,44,45,46,47].

TMSs are undesired components of cellulose and paper. They enter the paper together with cellulose, alum, rosin, additives, or other components in the paper-making process, or as impurities in the technological equipment. Scientific contributions devoted to the identification and determination of TMSs in finished paper or products of its processing are relatively rare.

Because the raw materials in paper production are not of the same origin and composition, and the production technologies differ on the basis of the requirements for paper characteristics, the content and type of TMSs can also vary significantly in the paper. To identify and determine metal elements in papers, two experimental techniques dominate, namely, inductively coupled plasma mass spectrometry (ICP-MS) [48] and energy-dispersive X-ray fluorescence [49]. Within forensic examination and dating of documents, Tanase et al. [50] determined several metal elements (Mn, Sr, Al, Mg, Ba, Fe, Zn, and Pb) in five paper brands. The results of this study show that trace element concentrations measured using ICP-MS can be used to discriminate white photocopy papers. Individual brands of paper could be distinguished by determining the concentration of a few elements.

In accordance with the need to prevent the toxic effect of packaging materials on food, the content of toxic metal elements in paper and cardboard food packaging was monitored [51,52]. Using inductively coupled plasma optical emission spectrometry, the authors identified the presence of Pb, Cd, Zn, Ni, Cu, Cr, Al, and Hg in the investigated paper and cardboard.

In addition to elemental analysis of paper brands, metal elements were also determined in technological byproducts and wastes [50,53,54]. An article published in 2008 [53] focusing on the environmental issues presented the results of an analysis of metallic elements in paper mill sludge. The researchers determined the concentration of the following elements: P, Ca, Na, K, Mg, S, Cu, Zn, Cd, Pb, Cr, Fe, Mn, Ni, Co, As, V, Ba, and Ti.

However, it should be remembered that not all metals are dangerous for paper. Zinc, magnesium, calcium, and barium compounds have all been used for deacidification. Of the TMSs, those able to participate in catalytic redox processes (Cu, Fe, V, Cr, and Mn) can be active in paper degradation. Unlike stoichiometric oxidation with oxygen and ROS or photochemical degradation, the activity of TMSs lies in their participation in catalytic processes, and they can, therefore, contribute significantly to the degradation of paper, even at trace concentration [45]. In studying the role of TMSs in paper oxidation, cellulose-containing systems or paper systems with added TMS, i.e., not the TMS naturally present in the paper, were usually used.

Model studies of cellulose oxidation in the presence of Fe^3+^ and Cu^2+^ ions have documented that, in a slightly alkaline environment, these ions play an important role in cellulose degradation, either as catalysts in the homolytic scission of the cellulose peroxide, which is formed by a free radical mechanism or through a donor–acceptor Lewis mechanism involving either the semiacetalic oxygen on the anhydroglucose unit or the β-glucosidic oxygen. In the first case, cleavage of a glucopyranose unit occurs; in the second one, depolymerization of the chain immediately takes place [12]. Bicchieri et al. [12] also found that the use of a reducing compound (she used a borane *tert*-butylamine complex) can impede oxidation catalyzed by metals, producing at the same time optical bleaching of paper. 

The catalytic effect of TMSs consists in the formation of reactive radicals, a typical example being the Fenton reaction (details can be found in [22,55,56,57]).

Fe^2+^ + H_2_O_2_ ⟶ Fe^3+^ + HO^•^ + OH^−^ (1)

Fe^3+^ + H_2_O_2_ ⟶ Fe^2+^ + HOO^•^ + H^+^ (2)

Knowledge of paper oxidation is important not only for paper as an information carrier and object of cultural heritage but also for other applications of paper. An example is the oxidation of paper impregnated with mineral oil, which is used as a part of insulation systems in power transformers [58]. Following the time development of the degree of polymerization and using microcalorimetry, the authors found that the reaction rate was not linear with oxygen concentration and the activation of oxidation (51 kJ/mol) differed from that of hydrolysis (110 kJ/mol).

## 5. Impact of Deacidification on the Stability of Alum-Containing Paper

Within the Kniha^SK^ project Consortium [59], an evaluation of the results on mass deacidification processes accumulated by relevant memory and academic institutions was performed. To evaluate and compare the effect of deacidification processes, acidic test paper containing alum-rosin sizing was chosen as a benchmark/model paper [11]. In all experiments, acidic test alum-rosin sized paper, grammage 45 g/m^2^, cold extract pH: 4.5–5.0, surface pH = 5.6 ± 0.3, containing bleached mechanical groundwood (55%), bleached sulfite pulp (20%), recycled fibers (15%), and clay (10%) was used. Test books containing 150 sheets of A5 paper underwent deacidification treatment.

To compare the efficacy of deacidification, the following mass-deacidification procedures, widely used in the world, were used: CSC Booksaver, IPC (Institut Politecnic del Campus de Terrassa, Terrassa, Spain); CSC Booksaver, PAL (Preservation Academy, Leipzig, Germany); Papersave Swiss, NCW (Nitrochemie Wimmis, Wimmis, Switzerland); Papersave, BI (Battelle Ingenieurtechnik, Eschborn, Germany); SoBu, Fürth; Papersave, ZFB (Zentrum für Bucherhaltung GmbH, Leipzig, Germany).

Paper degradation was simulated by accelerated aging of samples encapsulated in foil bags (Tenofan Al/116S) at 96 ± 2 °C for up to 15 days. Detailed results of evaluation according to various criteria and properties are described by Katuščák et al. [11].

According to the results of this comparative assessment, it can be said that deacidification processes are basic techniques used by memory institutions wishing to stabilize acidic paper (i.e., containing alum-rosin sizing) documents. The results showed that such papers can be successfully stabilized. Naturally, depending on the choice of deacidifying agent and technology, it is possible to achieve different stability and prolong the lifetime of paper documents.

**Figure 3:**
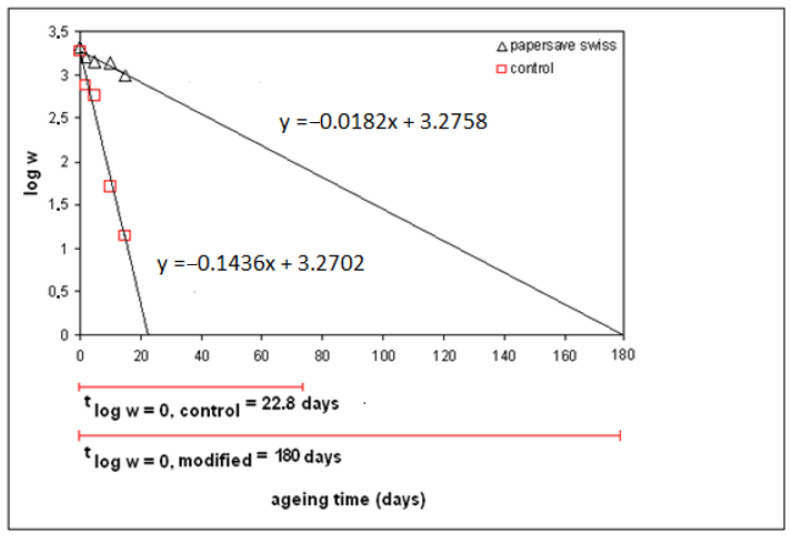
Evaluation of the lifetime for folding endurance for deacidification process Papersave Swiss.

The efficacy of individual deacidification methods was expressed in term of “increased mechanical permanence and lifetime prolongation”. This criterion of deacidification efficacy, proposed by the Library of Congress [60], reads as follows: “the rate at which paper loses strength upon accelerated aging at 90 °C/50% relative humidity (RH) for up to 30 days, shall be decreased by at least a factor of 3.0 when the logarithm of the folding endurance is plotted against time in days”. As an example, results achieved using the deacidification process Papersave Swiss are shown in Figure 3. When the permanence of the treated paper is increased by a factor of 300%, the books should keep their utility properties three times longer” [11,59,60]. The efficacy in terms of increased stability of mechanical properties and lifetime prolongation requirement was also adopted by the Kniha^SK^ consortium.

Time values for log *w* = 0 were calculated from the following linear equation:

(3) log w=A+B×t,

where *B* is the slope of the line (gradient) and *A* is the intercept on the log *w*-axis. The values were used for relative comparison of the efficacy of the deacidification process with regard to nontreated control samples. The lifetime of a paper terminates when the logarithm of folding endurance becomes zero (*t*_log *w* = 0_).

The coefficient of relative increase of the lifetime for folding endurance (*S*_τ(log *w* = 0)_) for the above example can be expressed as follows [61]:

(4) $$S_{\tau (\log w\;=\;0)}=\frac{t_{\log w\;=\;0,\;\mathrm{modified}}}{t_{\log w\;=\;0,\;\mathrm{control}}}=\frac{180}{22.8}=7.9.$$

Some of the results presenting the lifetime for folding endurance (S_τ(log *w* = 0)_) for deacidification processes are shown in Table 1. The order of deacidification processes quality was made according to Consortium Kniha^SK^, Bratislava, and The Library of Congress, Washington methods. The requirement was as follows: the coefficient of lifetime prolongation *S*_τ(log *w* = 0)_ must reach the value of 3 as a minimum [11,59]. With regard to the mechanical permanence, the most effective process is the Papersave Swiss and the least effective process is the CSC Booksaver, PAL.

## 6. Levels of pH and Alkaline Reserve Reached in Treating Alum-Containing Paper

All the deacidification processes were effective and led to pH values above 8. For any deacidification process, alkaline reserves in the range of 0.7–2.2% CaCO_3_ were created [62]. The highest pH (9.9) was reached in the CSC Booksaver, IPC process and it decreased gradually for the Papersave, BI (9.8), Papersave, ZFB (9.6), Papersave Swiss, NCW (8.9), Booksaver PAL (8.5), and SoBu, Fürth (8.2) processes. After 15 days of accelerated aging (96 °C, in bags), the CSC Booksaver process IPC led to the highest pH (9.5). The pH values for the other deacidification techniques were as follows: Papersave, BI processes (8.4); Papersave Swiss, NCW (7.9); SoBu, Fürth (7.8); Papersave, ZFB (6.5); CSC Booksaver, PAL (4.4).

The alkaline reserve, after modification by various deacidification procedures, was in the range 0.7–2.2% CaCO_3_. The highest value was reached in the process of CSC Booksaver, IPC (2.2% CaCO_3_) and it decreased gradually for the processes of Papersave, BI (1.8% CaCO_3_), Papersave Swiss, NCW (1.8% CaCO_3_), SoBu, Fürth (1.6% CaCO_3_), and Papersave, ZFB (1.4% CaCO_3_). The lowest alkaline reserve (0.7% CaCO_3_) was determined in the CSC Booksaver, PAL process. The pH value after deacidification by the tested newsprint processes corresponds to the requirements stipulated by ISO 9706 [57]. The newsprint after deacidification by the Papersave and CSC BookSaver Barcelona processes had a pH value of 8.9–9.9. ISO 9706 sets a minimum alkaline reserve of 2% CaCO_3_, and this value was achieved for newsprint deacidified by the CSC BookSaver process, Barcelona (2.2% CaCO_3_). Newsprint deacidified by the Papersave, Sobu and CSC Booksaver, PAL processes did not reach the required alkaline reserve value set by the ISO 9706 standard [63].

## 7. Stabilization and Conservation of Paper Documents

Maintenance, stabilization, and conservation of paper documents can be discussed from three perspectives on the basis of the type of degradation: hydrolytic degradation, oxidative degradation by ROS, including that catalyzed by a trace amount of TMS, and oxidative degradation caused by iron gall ink.

As mentioned above, paper can be stabilized against hydrolytic degradation using various deacidification agents and techniques [64,65,66,67,68]. An increase in pH leads, however, to a higher vulnerability to oxidative degradation. Deacidification also neutralizes acids formed at processes occurring during ink corrosion. It is not, however, sufficient to stop ink corrosion completely. Deacidification can be assessed as a very efficient tool of paper stabilization, but it is not an all-powerful tool.

Generally, the chemical compounds employed to prevent or suppress the oxidation of the paper—antioxidants—act in two principal modes: complexing catalytically active TMSs by incorporating them into redox nonreactive chelates or scavenging radicals and decomposing other oxidizing agents. Regarding the use of complexing agents, phytate (1,2,3,4,5,6-hexakis(dihydrogen phosphate)myo-inositol) is predominantly mentioned in the literature. This compound usually coordinates to the central atom by 2–6 oxygen atoms and forms chelates with relatively high stability constants [69,70,71]. The chelates formed do not act as catalysts for redox degradation processes.

The radical scavenging process may be illustrated using the reaction of hydroxyl radicals HO^•^ with iodide I^−^, which is an excellent radical scavenger.

I^−^ + HO^•^ ⟶ I^•^ + HO^−^ (5)

I^−^ + I^•^ ⟶ I_2_^•−^ (6)

In addition to the anion I^−^, anions Br^−^ and SCN^−^ also react with HO^•^. In alkaline (pH 9–10.5) solutions, the rate constants of the reaction are nearly diffusion-controlled [72]. In strongly alkaline solutions (pH 13), iodine I_2_ also participates in the decomposition of hydrogen peroxide; however, I^–^ in alkaline media does not decompose H_2_O_2_ [72].

I_2_ + 2 H_2_O_2_ ⟶ 2I^–^ + 2 H^+^ + O_2_ (7)

Laboratory investigations of the treatment of paper samples by antioxidants aimed at their stabilization (slowing down the rate of oxidative degradation caused by ROS) are based on the assumption of the validity of the Arrhenius rate constant/temperature relation. The results are obtained by accelerated aging at predetermined higher temperatures (usually ranging from 60 °C to 105 °C), time, and relative humidity, and are subsequently extrapolated to normal temperature. Antioxidants, mainly calcium phytate, KI, KSCN, and KBr, have been applied together with deacidification agents [46,64,73,74,75,76,77]. As measures of the effectivity of the used antioxidants, mechanical properties of paper, prolongation of the expected lifetime, yellowing, and the degree of polymerization have been monitored. According to the obtained results, it can be concluded that, due to the differences in paper kinds and the impacts of several ostensibly independent factors contributing to paper properties, it is not possible to predict and design the best general method of paper stabilization. In this area, much research needs to be done.

To monitor the effects of deacidification systems, different conditions of accelerated aging are used. However, there is still controversy as to whether these tests are sufficiently accurate to mimic natural aging [78]. An even greater debate arises in the use of stabilization systems that contain antioxidants. The reason is that the stabilization system naturally also affects the comparison of accelerated and natural aging. The error in the results and conclusions are related to the degree of accelerated tests, especially with regard to oxidation reactions. This is because oxidation cannot be accelerated via the same mechanism as under natural conditions. Accelerated aging is not the same in the individual phases; this applies in particular to the absorption of oxygen. The comparison is relatively accurate only in those conditions where the extrapolated temperature lies in the region where the same degradation mechanism predominates. Some antioxidants, antiozonants, flee rapidly at elevated temperatures, and the result is that their accelerating effect weakens with accelerated aging [79]. Under normal conditions, aging is involved in free radical reactions or direct oxidation.

If the antioxidation stabilization system contains more than one inhibitor, additivity usually occurs, and the overall effect is often in line with the sum of the effects of the individual antioxidants (synergism). Conversely, if the effect is weakened, we speak of antagonism. When antioxidants act via the same mechanism but differ in their efficiency, we speak of homosynergism. Heterosynergism in a mixture of antioxidants describes a situation when they act via different mechanisms [80]. Synergism was reported in a combination of compounds that disrupt chain oxidation and degrade peroxides [79,80]. The development of specific stabilization systems has focused mainly on the field of deacidification systems. So far, various types of antioxidant chemicals, substances acting synergistically with antioxidants, and metal deactivators have been gradually applied on a laboratory scale. Naturally, the application of such systems has specific requirements: high resistance of the stabilizer to oxygen oxidation and the formation of color conversion products, inertness to the substrate components, and, of course, sufficient solubility in the deacidifying agent carrier. The complexity of the selection is increased by the requirement for physiological safety, inertness, and respect for the aesthetic and physical properties of the substrate [79].

To evaluate and compare the effect of the oxidation process, various methods can be used. In the oxidation of substances of organic origin, the measurement of oxygen absorption during oxidation is often used. This method of studying the rate of oxidation makes it possible to compare the oxidation over a relatively wide temperature range in the presence of initiators or inhibitors. Moreover, the method of weight gain of oxidized products, volumetric methods that determine changes in oxygen volume at constant pressure, and manometric methods for determining oxygen absorption during oxidation at changed pressure in the system can be employed. It is also important to know the chemical composition of oxidation products. This knowledge of the formation of products during the oxidation process allows determining the kinetics of this process and the influence of various factors on the oxidation process: peroxy compounds and radicals ROO^•^, compounds with alcohol group, epoxy compounds, carbonyl, carboxyl groups and esters, or double bonds.

## 8. Conclusions

In Section 1, four questions were formulated, which we addressed in the subsequent sections of the document. According to the current understanding of the causes and mechanisms of paper degradation, it is clear that deacidification alone cannot suffice to slow down or even stop degradation, and that slowdown of oxidation will also have to be considered including the impact of different types of transition metal species in paper and ink.

Deacidification processes are a technology used by memory institutions making it possible to stabilize and achieve a sufficient alkaline reserve and pH, depending on the type of deacidification system, as well as for acidic paper documents containing alum-rosin sizing. The homogeneity of the distribution of alum on the surface of the paper and its volume depends on the papermaking technology. One could expect that, if, in the papermaking process, the alum and rosin together with the cellulose form part of the cellulosic solution, the rosin and alum should be distributed homogeneously in the paper. As documented by the authors of [4,5], this is not the case. The reason lies in the internal structure of paper and specific adsorption of alum and rosin on the fibers. It should be noted that a detailed analysis of the distribution of alum in the cross-section of paper has not yet been performed. In the case of a surface application of a mixture of alum and rosin, the distribution is homogeneous, but local damage to the layer occurs during the use of the paper. Sites with different alum contents have different degradability. However, the definitive answer to question 4 requires several studies to be performed in the future.

On the basis of current scientific knowledge and its application in practice, it can be assumed that new paper stabilization technologies will be developed combining the suppression or at least slowing down of its hydrolytic, oxidative, and biological degradation. The methods of stabilization will differ depending on whether they concern the stabilization of individual documents of historical significance (edicts, founding documents, charters, bulls, or decrees in archives and museums) or documents of mass production (books, journals, or newspapers in libraries).

## Figures and Tables

**Table 1:** Evaluation of the lifetime for folding endurance (*S*_τ(log *w* = 0)_) for deacidification processes [11,59].

Process, Aging 96 °C, Up to 15 days	A	B	*t*_(log *w* = 0)_	*S*_τ(log *w* = 0)_
Papersave Swiss, NCW	3.27579	−0.0182	180	7.9
Papersave, BI	3.31894	−0.02214	150	6.6
SoBu, Fürth	3.11813	−0.02937	106.2	4.7
Papersave, ZFB	3.26621	−0.03121	104.7	4.6
CSC Booksaver, IPC	3.20072	−0.05512	58.1	2.6
CSC Booksaver, PAL *	3.3919	−0.18753	18.1	1.3
Control 1	3.27024	−0.14361	22.8	1.0
Control 2 *	2.88573	−0.20292	14.2	1.0

* The sample of unmodified paper (control 2) was used for process CSC Booksaver, PAL.
